# A acute pontine infarction with one-and-a-half syndrome as the manifestation: A case report

**DOI:** 10.1097/MD.0000000000039144

**Published:** 2024-08-02

**Authors:** Jing Fu, Hui Yang, Shasha Liao

**Affiliations:** a The College of Clinical Medicine 2, Guizhou University of Traditional Chinese Medicine, Guiyang, Guizhou Province, China; b The Second Affiliated Hospital of Guizhou University of Traditional Chinese Medicine, Guiyang, Guizhou Province, China.

**Keywords:** acupuncture, acute pontine stroke, internuclear ophthalmoplegia, neuroimaging, one-and-a-half syndrome

## Abstract

**Rationale:**

Sudden ocular dyskinesia is usually associated with ophthalmic diseases and rarely with cerebrovascular diseases. This is a rare case of a patient with a sudden onset of ocular dyskinesia due to occlusion of the anterior inferior cerebellar artery and the spiral modiolar artery. This article describes eye movement disorders associated with cerebrovascular disease, aiming to improve our understanding of cerebrovascular diseases and improve the ability of early diagnosis and differential diagnosis.

**Patient concerns:**

A 52-year-old man presented with acute pontine cerebral infarction 2 days before presentation. The main symptoms were the inability to adduct and abduct the left eyeball, the ability to abduct but not adduct the right eyeball, and horizontal nystagmus during abduction. Cranial computed tomography in our emergency department suggested cerebral infarction, and magnetic resonance imaging examination after admission confirmed the diagnosis of acute pontine cerebral infarction.

**Diagnosis:**

This patient was ultimately diagnosed with acute pontine cerebral infarction.

**Interventions:**

He received aspirin, clopidogrel, and butylphthalide, as well as acupuncture and Chinese herbal medicine.

**Outcomes:**

After 10 days of treatment, the patient’s paralysis of the eye muscles improved significantly.

**Lessons:**

Eye movement disorders are sometimes an early warning sign of impending vertebrobasilar ischemic stroke. Patients with acute ischemic stroke who have early detection of oculomotor disturbances should be promptly imaged, as missed diagnosis may lead to serious consequences or even death. It provided us with a new diagnostic idea.

## 1. Introduction

Internuclear ophthalmoplegia (INO), also known as medial longitudinal fasciculus syndrome, is divided into anterior INO, posterior INO, and one-and-a-half syndrome according to the location of damage to the medial longitudinal fasciculus. One-and-a-half syndrome, first reported by Fisher in 1967, is caused by pontine tegmentum lesions on one side along with the involvement of the parapontine midline reticular structure, the lateral pontine optic center, or the abducens nerve nucleus. And present with involvement in the contralateral crossed contact ipsilateral oculomotor caused by medial longitudinal fasciculus of the medial rectus muscle. The main clinical manifestations are: when both eyes are gazing toward the side of the lesion, the ipsilateral eyeball cannot abduct, and the contralateral eyeball cannot adduct (gaze palsy) when both eyes are gazing toward the contralateral side of the lesion, the ipsilateral eye cannot abduct, but the contralateral eye can abduction (INO), abduction is often accompanied by horizontal nystagmus, When appearing simultaneously, the eye on the side of the lesion showed limitation in adduction and abduction while the eye on the opposite side of the lesion presented limitation in adduction. This syndrome is clinically rare, and acute pontine infarction in which one-and-a-half syndromes are the only clinical manifestations is even rarer. A typical case treated in our hospital is reported below.

## 2. Case presentation

**Figure 1.**
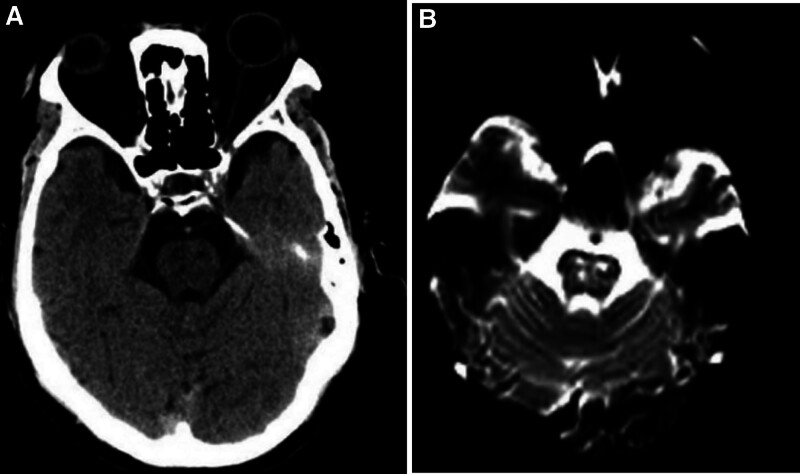
The brain computed tomography images show multiple small nodular low-density shadows in the pons (A) and the brain magnetic resonance image reveals punctate high-density shadows in the left pontine tegmentum (B).

The patient, a 52-year-old male, was admitted to the emergency department due to “sudden-onset dizziness accompanied by double vision for 2 days.” The patient suddenly became dizzy without obvious triggers and presented with a persistent feeling of drowsiness with the onset of double vision. Urgent examination of the brain computed tomography (Fig. 1A) showed multiple lacunar lesions in the pons and bilateral basal ganglia accompanied by softening and deep ischemic changes in the white matter of the brain. He had a cerebral infarction and hypertension lasting >1 year. The history of “cerebral infarction” has left the symptoms of nonfluent speech, and has no standardized secondary preventive treatment for cerebrovascular disease. He had been smoking 40 cigarettes a day for 50 years and drinking 100 mL alcohol per time occasionally. He is a quick-tempered man and his diet is rich in fat and sweet. He denies any history of drug allergies and has no family genetic history. The neurological examination showed that the left eye is restricted adduction and abduction, while the right eye is restricted adduction and an abducting horizontal nystagmus was observed in the right eye. The right Babinski sign and Chaddock signs were positive and the remaining neurological examinations showed no abnormality. The neck blood vessels color Doppler ultrasound demonstrating multiple plaques formatted carotid arteries atherosclerotic bilaterally. The cranial magnetic resonance imaging (Fig. 1B) shows a high signal lesion next to the midlines of the bilateral pontine tegmentum, which is considered an acute pontine infarction. Computed tomography angiography of the neck and head revealed noncalcified plaques formation on the wall of the A1 segment of the right anterior cerebral artery with severe lumen stenosis (71%) and noncalcified plaques were seen on the wall of the P1 segment of the right posterior cerebral artery with severe lumen stenotic. severe stenosis of the left internal carotid artery.

After admission, the treatment and nursing measures were implemented using an integrative Chinese and Western medicine mode for the patient. Given aspirin and clopidogrel to antiplatelet aggregation, antithrombosis, butylphthalide, and urinary kallidinogenase to improve brain tissue energy metabolism. Moreover, using acupuncture for resolving phlegm, extinguishing wind, and dredging collaterals combined with Chinese patent medicine for promoting blood circulation, removing blood stasis and unblocking meridians, and activating collaterals as adjuvant therapy. After 11 days, the symptoms of visual ghosting improved and the left eye was fully abducted and adducted. The abduction movements of the right eyeballs without horizontal tremor were also observed. Standardized therapeutic regimens such as drugs, out-of-hospital rehabilitation, and secondary prevention of cerebrovascular disease have been guided for the patient.

## 3. Discussion

The medial longitudinal fasciculus, is located in the paramedian portion of the upper brainstem tegmentum. Anterior INO: When one side of the medial longitudinal fasciculus is damaged, the fibers from the lateral optic center of the pons cannot pass through the contralateral medial longitudinal fasciculus to the medial rectus muscle innervated by the oculomotor nucleus.^[1]^ When the eyes move toward the healthy side, the eyeball on the side cannot adduct, but abduction is normal. When the eyeball on the unaffected side is adducted, diplopia occurs with horizontal tremor, and the convergence reflex is normal. Posterior INO: The fiber damage between the pontine optic center and the abducens nerve nucleus on one side prevents the abducens nerve nucleus fibers from innervating the ipsilateral extraocular rectus muscle. When both eyes move in the same direction, the affected side cannot abduct. The vergence reflex was normal. One-and-a-half syndrome is also called pontine paralytic exotropia: the fibers between the pontine optic center on one side and the abducens nerve nucleus and the medial longitudinal fasciculus from the pontine optic center on one side to both sides are damaged, and the eyeball on the affected side cannot adduct or abduct, the unaffected eyeball cannot adduct (half), and is accompanied by horizontal tremor when abducting, but the convergence reflex of both eyes is normal. The causes of INO are complex and are more common in multiple sclerosis, vascular diseases, brainstem inflammation, or tumors. Bilateral lesions are more common in younger patients with multiple sclerosis, and unilateral lesions in the elderly are mostly caused by vascular factors.^[2]^

**Figure 2.**
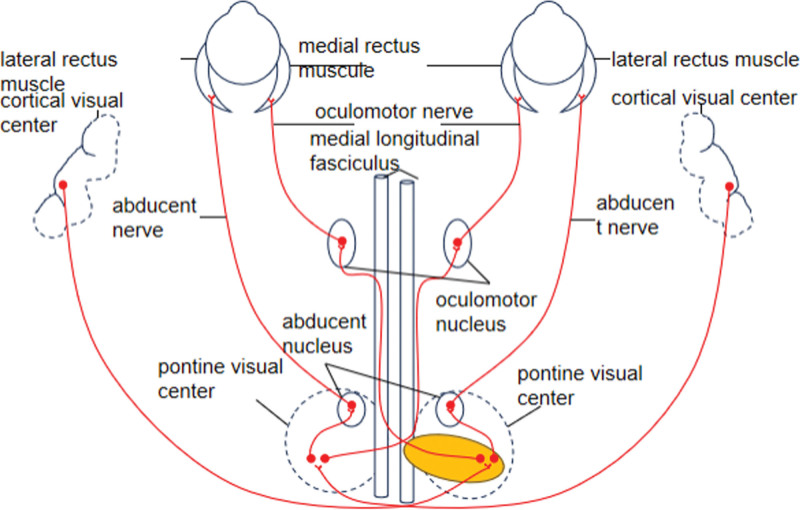
Schematic diagram of the impairment of horizontal movement disorders in the patient’s eyeball (yellow is the infarct area).

The patient in this case is a middle-aged male who complained of dizziness and double vision. The left eye was limited in adduction and abduction, and limitation of adduction of the right eye with horizontal tremor occurs during abduction movement. And there was no other neurological injury symptom detected on physical examination. An imaging examination shows an abnormality in the pontine. It is considered as nidus responsible for the symptoms and the location of responsible lesions is consistent with the anatomical positioning of the medial longitudinal fasciculus in the left pontine tegmentum. Because the lesion damages the ascending fibers of the medial longitudinal fasciculus that have crossed and innervated the medial rectus muscle on the unaffected side (Fig. 2), the symptoms are as follows: when the eyes look toward the contralateral side of the lesion, the affected side The eyeball cannot adduct, but the unaffected eyeball can abduct, with tremor during abduction, the tremor may be related to Hering’s law.^[3,4]^

The lateral rectus on the side of the lesion fails to adduct the eye normally on attempted horizontal gaze to the same side and the ipsilateral abducens nerve nucleus also be injured by the lesion. Second, the lateral rectus muscle fibers innervated by the lateral rectus muscle fibers fail to abduct the affected eyeball, the eye normally on attempted horizontal gaze to the same side. Therefore, the patient was diagnosed with one-and-a-half syndrome. The supranuclear pathways for convergence control are normal, thus the medial rectus muscle of the affected side of the eyeball is more likely to be impaired by the ascending fibers of the medial longitudinal fasciculus. The unimpaired superior colliculus illustrates the vertical eye movement is normal. Meanwhile, the surrounding tissue were not damaged by the lesion and the patient did not show facial paralysis, dysarthria, or paresthesia, etc. The main clinical manifestation of the disease was acute onset and the patient has a history of cerebral infarction, and hypertension, so we diagnosed cerebrovascular disease with small artery occlusion based on acute onset, clinical signs, and magnetic resonance imaging findings. The main blood supply from the basilar artery parapontine long circumflex artery via the anterior inferior cerebellar artery and the spiral modiolar artery. One-and-a-half syndrome, a rare disease characterized by clinical and radiological findings, is easy to miss diagnosis and misdiagnosis. Therefore, it needs to memorize the anatomical location of the medial longitudinal fasciculus and the ocular characteristics of INO. More importantly, the opportune detection of those lesions and timely treatment offers the possibility of a cure. The patient did not present to the hospital in time for the initial onset of vertigo symptoms, resulting in missed thrombolysis, limited treatment options available to the physician, and ultimately incomplete improvement of symptoms.

## Acknowledgments

We are very grateful for the support of the Second Affiliated Hospital of Guizhou University of Traditional Chinese Medicine and the teachers of Guizhou University of Traditional Chinese Medicine. (Informed consent has been obtained from all informants).

## Author contributions

**Conceptualization:** Jing Fu.

**Data curation:** Jing Fu.

**Formal analysis:** Jing Fu.

**Investigation:** Jing Fu.

**Methodology:** Jing Fu.

**Resources:** Jing Fu.

**Funding acquisition:** Hui Yang.

**Supervision:** Hui Yang, Shasha Liao.

**Writing—original draft:** Jing Fu.

**Writing—review and editing:** Jing Fu.
